# Determination of cepharanthine in rat plasma by LC–MS/MS and its application to a pharmacokinetic study

**DOI:** 10.1080/13880209.2017.1328446

**Published:** 2017-05-19

**Authors:** Yingbin Deng, Weijun Wu, Sunzhi Ye, Wei Wang, Zhiyi Wang

**Affiliations:** Department of Emergency Medicine, The Second Affiliated Hospital and Yuying Children’s Hospital of Wenzhou Medical University, Wenzhou, China

**Keywords:** Absorption, bioavailability, P-gp

## Abstract

**Context:** Cepharanthine (CPA) has been reported to possess a wide range of pharmacological activities.

**Objective:** This study investigates the pharmacokinetic characteristics after oral or intravenous administration of CPA by using a sensitive and rapid LC–MS/MS method.

**Materials and methods:** A sensitive and rapid LC–MS/MS method was developed for the determination of CPA in Sprague–Dawley rat plasma. Twelve rats were equally randomized into two groups, including the intravenous group (1 mg/kg) and the oral group (10 mg/kg). Blood samples (250 μL) were collected at designated time points and determined using this method. The pharmacokinetic parameters were calculated.

**Results:** The calibration curve was linear within the range of 0.1–200 ng/mL (*r* = 0.999) with the lower limit of quantification at 0.1 ng/mL. After 1 mg/kg intravenous injection, the concentration of CPA reached a maximum of 153.17 ± 16.18 ng/mL and the *t*_1/2_ was 6.76 ± 1.21 h. After oral administration of 10 mg/kg of CPA, CPA was not readily absorbed and reached *C_max_* 46.89 ± 5.25 ng/mL at approximately 2.67 h. The *t*_1/2_ was 11.02 ± 1.32 h. The absolute bioavailability of CPA by oral route was 5.65 ± 0.35%, and the bioavailability was poor.

**Discussion and conclusions:** The results indicate that the bioavailability of CPA was poor in rats, and further research should be conducted to investigate the reason for its poor bioavailability and address this problem.

## Introduction

Cepharanthine (CPA), a biscoclaurine alkaloid isolated from *Stephania cepharantha* Hayata (Menispermaceae), has been reported to possess a wide range of pharmacological activities (Azuma et al. 2006; Chea et al. 2007; Desgrouas et al. 2014). CPA has been widely used for the treatment of many acute and chronic diseases, such as venomous snakebite, alopecia areata, exudative otitis media, and endotoxic shock (Furusawa & Wu 2007; Kusaka et al. 2011; Rogosnitzky & Danks 2011). CPA also exhibits potent anticancer activity *in vitro* for different cancer cells (Biswas et al. 2006; Harada et al. 2009; Seubwai et al. 2010; Ikeda et al. 2011; Chen et al. 2012). Some research articles (Ikeda et al. 2005; Seo et al. 2009; Li et al. 2011; Zahedi et al. 2011; Han et al. 2014) have reported that CPA could modulate the activity of P-glycoprotein (*P-gp*) through downregulation of the expression MDR1, and therefore, combined with other drugs, it could be used for overcoming the multidrug resistance phenomenon in cancer therapy.

Due to the potent pharmacological activities of CPA, it is of great significance to investigate the pharmacokinetic properties of CPA. Hao et al. (2010) have developed a sensitive and reliable LC–MS/MS method to determine the concentration of CPA in human plasma, and investigate the pharmacokinetics profiles of CPA after single intravenous administration of 50 mg CPA. However, to the best of our knowledge, there is little data available regarding the oral bioavailability of CPA. Investigating the bioavailability of CPA in rats will be helpful for the development of preparations and pharmacological investigations, and it could also provide a basis for the rational drug use and prediction of drug toxicity reaction. To enhance the development potential of CPA, there is an urgent need to investigate the pharmacokinetic profiles of CPA, especially its bioavailability characteristics.

This study investigates the pharmacokinetic characteristics after oral or intravenous administration of CPA by using a sensitive and rapid LC–MS/MS method.

## Materials and methods

### Chemicals and reagents

CPA (purity >98%) and rutin (purity >98%) were purchased from the National Institute for the Control of Pharmaceutical and Biological Products (Beijing, China). Acetonitrile and methanol were purchased from Fisher Scientific (Fair Lawn, NJ). Formic acid was purchased from Anaqua Chemicals Supply Inc. Limited (Houston, TX). All other chemicals were of analytical grade or better.

### Instrumentation and conditions

The analysis was performed on an Agilent 1290 series liquid chromatography system (Agilent Technologies, Palo Alto, CA) and an Agilent 6470 triple-quadruple mass spectrometer (Agilent Technologies, Santa Clara, CA). Chromatographic separation of CPA and rutin was performed on Waters X-Bridge C18 column (3.0 × 100 mm, i.d.; 3.5 μm, Milford, MA) at room temperature (25 °C). The mobile phase was water (containing 1 mM ammonium formate and 0.05% formic acid) and methanol (35:65, v:v) with isocratic elution at a flow rate of 0.3 mL/min, and the analysis time was 1.5 min. The injection volume was 2 μL and the auto-sampler temperature was maintained at 25 °C.

The mass scan mode was positive MRM mode, and the mass parameters were optimized using Optimizer software. The precursor ion and product ion are *m/z* 607.3 → 365.3 for CPA and *m/z* 610.9 → 355.9 for rutin (internal standard), respectively. The collision energy for CPA and rutin was 30 and 25 eV, respectively. The MS/MS conditions were optimized as follows: fragmentor, 160 V; capillary voltage, 4 kV; Nozzle voltage, 500 V; nebulizer gas pressure (N_2_), 40 psig; drying gas flow (N_2_), 10 L/min; gas temperature, 350 °C; sheath gas temperature, 400 °C; sheath gas flow, 11 L/min.

### Pharmacokinetic study

#### Animals

Male Sprague–Dawley (SD) rats weighing 220–250 g were provided by the Experimental Animal Center of the Shandong University (Shandong, China). Rats were bred in a breeding room at 25 °C, 60 ± 5% humidity, and a 12 h dark–light cycle. Tap water and normal chow were given *ad libitum*. All the experimental animals were housed under the above conditions for a five day acclimation, and were fasted overnight before the experiments. The study was approved by the Animal Care Committee of the Shandong University (Shandong, China) and performed in accordance with the Guide for the Care and Use of Laboratory Animals (National Research Council 2016).

### *In vivo* pharmacokinetic study

Rats were fasted for 12 h with free access to water prior to the pharmacokinetic study. Twelve rats were equally randomized to two groups and were treated as followings: intravenous injection of CPA in normal saline was administrated through lateral tail vein at a dose of 1 mg/kg. An oral gavage of CPA dissolved in normal saline containing 0.5% methylcellulose solution was given to rats at a dose of 10 mg/kg. Blood samples (250 μL) were collected into a heparinized tube via the *oculi chorioideae* vein at 0.083, 0.167, 0.33, 0.5, 1, 2, 4, 6, 8, 12, 24 and 48 h, respectively. After centrifugation at 4000 rpm for 10 min, plasma samples were obtained and frozen at –40 °C until analysis.

### Plasma sample preparation

Plasma sample (100 μL) was spiked with 10 μL of the rutin (100 ng/mL), and then the mixture was extracted with acetonitrile (190 μL) by vortexing for 1 min. After centrifugation at 12,500 rpm for 10 min, the supernatant was injected into the LC–MS/MS for the determination.

### Preparation of standard and quality control samples

A stock solution of CPA was prepared in acetonitrile at a concentration of 2 mg/mL. The stock solution of rutin was prepared in acetonitrile at a concentration of 1 μg/mL. Calibration standard samples for CPA were prepared in blank rat plasma at concentrations of 0.1, 0.5, 1, 5, 10, 50, 100 and 200 ng/mL. The quality control (QC) samples were prepared at low (0.2 ng/mL), medium (10 ng/mL) and high (150 ng/mL) concentrations in the same way as the plasma samples for calibration, and QC samples were stored at –40 °C until analysis.

### Method validation

The method validation assay was performed according to the United States Food and Drug Administration (FDA) guidelines.

### Specificity

Specificity was investigated by comparing the chromatograms of six different batches of blank rat plasma with the corresponding spiked plasma to monitor interference of endogenous substances and metabolites.

### Linearity and sensitivity

To obtain the calibration curve, seven concentrations of the calibration standard were processed and determined as described above. The linearity of calibration curves was constructed by plotting peak area ratios (*y*) of the analyte to rutin against the nominal concentration (*x*) of analyte with weighted (1/*x*^2^) least square linear regression. The lower limit of detection (LLOD) and lower limit of quantification (LLOQ) were determined as the concentration of the analyte with a signal-to-noise ratio at 3 and 10, respectively.

### Precision and accuracy

The intra-day precision and accuracy of the method were confirmed by determining QC samples at three different concentrations five times on a single day, and the inter-day precision and accuracy were assessed by determining the QC samples over three consecutive days. For each concentration, five replicates were prepared. Relative standard deviation (RSD) and relative error (RE) were used to express the precision and accuracy, respectively.

### Extraction recovery and matrix effect

The extraction recovery was assessed by comparing peak areas obtained from extracted spiked samples with those originally spiked in the blank plasma samples. The matrix effect was evaluated by comparing the peak areas of the post-extracted spiked QC samples with those of corresponding standard solutions. These procedures were repeated for five replicates at three QC concentration levels.

### Stability

For sample stability, three levels of QC samples were determined under different conditions, including short-term stability at room temperature for 24 h, long-term stability at –40 °C for 30 days and three freeze–thaw cycles at –40 °C.

### Data analysis

The pharmacokinetic parameters, including area under the plasma concentration–time curve (*AUC*), maximal plasma concentration (*C_max_*), the time for maximal plasma concentration (*T_max_*) and mean residence time (*MRT*) were calculated using DAS 3.0 pharmacokinetic software (Chinese Pharmacological Association, Anhui, China).

## Results and discussion

### Sample preparation

Due to the complex nature of plasma, a sample pretreatment procedure is often needed to remove protein and potential interferences before LC–MS/MS analysis. In this study, protein precipitation, solid phase extraction (SPE) and liquid–liquid extraction were investigated to achieve good resolution and high recovery of analyte from spiked biologic matrices. The recovery of direct protein precipitation method using acetonitrile (88.7 ± 5.8) was better than SPE (82.4 ± 4.7) and liquid–liquid extraction method (76.5 ± 4.2), and the direct protein precipitation method was much easier to operate. Finally, direct protein precipitation method using acetonitrile was selected for biological sample preparation.

### Chromatography and mass spectrometry

**Figure 1.**
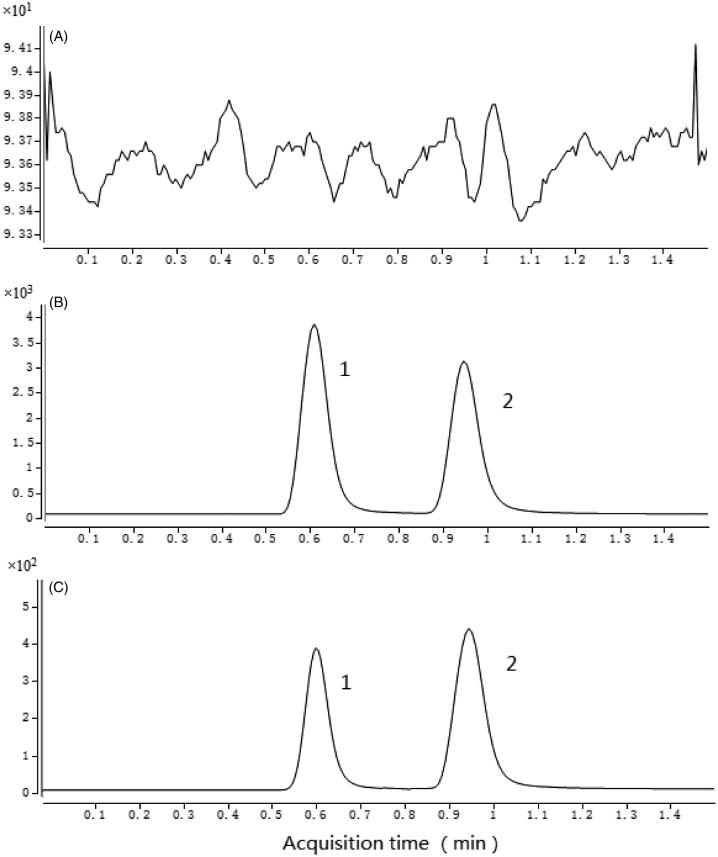
Chromatograms of blank plasma (A); plasma spiked with CPA and rutin (B); rat plasma sample obtained 10 h after oral administration of CPA (C). 1: rutin; 2: CPA.

The optimized mass transition ion-pairs for quantification, including precursor and product ions, were *m/z* 607.3 → 365.3 for CPA and *m/z* 610.9 → 355.9 for rutin, respectively. Blank plasma, plasma spiked with CPA and rutin are shown in Figure 1. No interference substances were observed at the retention time of CPA and rutin in plasma samples.

### Method validation

The standard curve for CPA in plasma was linear in the concentration range of 0.1–200 ng/mL (*y* = 0.295*x* + 0.119, *r* = 0.999). The LLOQ and LLOD were 0.1 and 0.038 ng/mL, respectively.

Intra-day and inter-day precision and accuracy were determined by measuring six replicates of QC samples at three concentration levels in rat plasma. The precision and accuracy data are shown in Table 1. These results demonstrated that the precision and accuracy values were well within an acceptable range of 15%.

**Table 1.** The intra-day and inter-day precision and accuracy of cepharanthine in plasma samples.

		Intra-day			Inter-day		
Analyte	Plasma samples (ng/mL)	Concentration measured (ng/mL)	Precision (%, RSD)	Accuracy (%, RE)	Concentration measured (ng/mL)	Precision (%, RSD)	Accuracy (%, RE)
Cepharanthine	0.2	0.19	6.87	–5	0.22	5.64	10
	10	10.57	5.59	5.70	9.14	7.52	–8.60
	150	141.25	6.38	–5.83	161.25	8.24	7.50

The mean extraction recoveries determined using three replicates of QC samples at three concentration levels in rat plasma were 87.8 ± 6.5%, 92.4 ± 5.7% and 85.6 ± 6.5% for 0.2, 10 and 150 ng/mL, respectively.

For ionization, the peak areas of CPA after spiking evaporated plasma samples at three concentration levels were comparable to those of similarly prepared aqueous standard solutions (ranging from 91.6% to 103.8%), suggesting that there was no measurable matrix effect that interfered with CPA determination in rat plasma.

The stability of CPA in plasma was evaluated by analysing three replicates of QC samples containing 0.2, 10 and 150 ng/mL CPA after short-term storage (25 °C, 24 h), long-term cold storage (–40 °C, 30 days) and within three freeze (–40 °C)–thaw (room temperature) cycles. As shown in Table 2, all of the samples displayed 90–110% recoveries after various stability tests. Taken together, the above results show that a rapid, simple and sensitive method for analysing CPA in rat plasma samples.

**Table 2.** Stability of CPA in plasma samples (*n* = 3).

		Stability (%, RE)		
Analyte	Plasmasamples (ng/mL)	Short-term (24 h at room temperature)	Long-term (30 days at –40 °C)	Three freeze (–40 °C)–thaw (room temperature) cycles
CPA	0.2	4.68	–7.39	7.68
	10	7.45	7.25	6.87
	150	5.36	6.98	9.77

Yasuda et al. (1989) have also investigated the pharmacokinetic characteristics of CPA using a sensitive HPLC method, and however, the SPE procedure was very tedious, and the plasma volume used was large, which restricted its clinical use. Hao et al. (2010) have reported a sensitive and reliable LC–MS/MS method for the determination of pharmacokinetic profiles of CPA after intravenous administration of CPA, the limit of quantification was 0.5 ng/mL, and the analysis time was 5 min. The LC–MS/MS method developed in this study was much simple (direct protein precipitation), sensitive (LLOQ at 0.1 ng/mL) and rapid (1.5 min analysis time) compared with these methods. In addition, we have also investigated the pharmacokinetics profiles of CPA after oral administration, which was not studied previously.

### Pharmacokinetic studies

**Figure 2.**
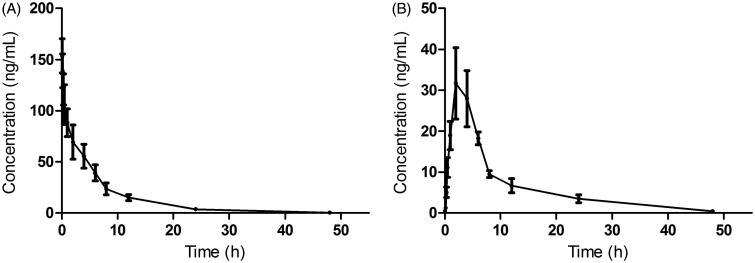
The pharmacokinetic profiles of CPA in rats after intravenous administration of CPA (A) at dosage of 1 mg/kg and oral administration of CPA (B) at 10 mg/kg.

The validated analytical method was employed to study the pharmacokinetic behaviours of CPA in rats. The mean plasma concentration–time curves of CPA after intravenous or oral administration of CPA are presented in Figure 2.

The pharmacokinetic parameters were calculated using the noncompartmental method with DAS 3.0 pharmacokinetic software (Chinese Pharmacological Association, Anhui, China). The pharmacokinetic parameters are shown in Table 3. The oral bioavailability was calculated by using *AUC_oral/dose_* divided by *AUC_iv/dose_*.

**Table 3.** Pharmacokinetic parameters of CPA in rats after intravenous (1 mg/kg) or oral administration (10 mg/kg) of CPA (*n* = 6, mean ± SD).

Parameters	Intravenous	Parameters	Oral
*T_max_* (h)	–	*T_max_* (h)	2.67 ± 1.16
*C_max_* (ng/mL)	153.7 ± 16.18	*C_max_* (ng/mL)	46.89 ± 5.25
*t*_1/2_ (h)	6.76 ± 1.21	*t*_1/2_ (h)	11.02 ± 1.32
AUC_(0–_*_t_*_)_ (ng·h/mL)	717.81 ± 158.35	AUC_(0–*t*)_ (ng·h/mL)	406.63 ± 62.57
AUC_(0–inf)_ (ng·h/mL)	721.80 ± 160.76	AUC_(0–inf)_ (ng·h/mL)	422.26 ± 66.91
MRT_(0–_*_t_*_)_ (h)	7.04 ± 0.49	MRT_(0–*t*)_ (h)	10.49 ± 0.62
MRT_(0–inf)_ (h)	7.30 ± 0.51	MRT_(0–inf)_ (h)	12.45 ± 1.20
Clz (L/h/kg)	1.431 ± 0.31	Clz/F (L/h/kg)	24.08 ± 2.42
Vz (L/kg)	13.79 ± 1.76	Vz/F (L/kg)	381.37 ± 61.63

*C_max_*: peak plasma concentration; *T_max_*: the corresponding time to reach *C_max_*; *t*_1/2_: the terminal elimination half-life; MRT: mean residence time; AUC_0–_*_t_*: AUC_(0–inf)_: the areas under the plasma concentration–time curve from time zero to the last quantifiable time-point and to infinity; CLz: clearance; Vz: apparent volume of distribution.

After 1 mg/kg intravenous injection, the concentration of CPA reached the peak plasma concentration of 153.17 ± 16.18 ng/mL and the *t*_1/2_ was 6.76 ± 1.21 h. After oral administration of 10 mg/kg of CPA, CPA was not readily absorbed and reached *C_max_* of 46.89 ± 5.25 ng/mL at approximately 2.67 h. The *t*_1/2_ was 11.02 ± 1.32 h. These results revealed that CPA was distributed and eliminated slowly in rats. The absolute bioavailability of CPA by oral route was 5.65 ± 0.35%, which demonstrated that the drug has poor absorption in rats through oral administration.

As reported by Hirai et al. (1995), CPA was a substrate of *P-gp*, and CPA was transported by *P-gp* in intestine. Therefore, we suggested that *P-gp* might hinder its absorption in intestine, and lead to its poor bioavailability in rats (Miao et al. 2016; Wang et al. 2016). The low metabolic stability in liver might also be a reason for its poor bioavailability. Further researches should be conducted to investigate the role of *P-gp* in the transport of CPA in intestine and its effect on the absorption of CPA, the metabolism in liver, while improving its oral bioavailability. As the species differences in the properties of metabolism enzymes between rats and human, it is essential to investigate the pharmacokinetics profiles of CPA in human.

## Conclusions

In conclusion, a sensitive and rapid LC–MS/MS method has been developed and successfully applied to determine the concentration of CPA in rat plasma. Using this method, the pharmacokinetic characteristics of CPA in rats were investigated, and the results indicated that the bioavailability of CPA was poor in rats. Further research should be conducted to investigate the reason for its poor bioavailability and address this problem.
